# Safety and Efficacy of the Common Vaccines against COVID-19

**DOI:** 10.3390/vaccines10040513

**Published:** 2022-03-25

**Authors:** Ying Liu, Qing Ye

**Affiliations:** Department of Clinical Laboratory, The Children’s Hospital, Zhejiang University School of Medicine, National Clinical Research Center for Child Health, National Children’s Regional Medical Center, Hangzhou 310057, China; 6521111@zju.edu.cn

**Keywords:** COVID-19, SARS-CoV-2, safety, efficacy, prime-boost strategies

## Abstract

The worldwide pandemic of coronavirus disease 2019 (COVID-19) has imposed a challenge on human health worldwide, and vaccination represents a vital strategy to control the pandemic. To date, multiple COVID-19 vaccines have been granted emergency use authorization, including inactivated vaccines, adenovirus-vectored vaccines, and nucleic acid vaccines. These vaccines have different technical principles, which will necessarily lead to differences in safety and efficacy. Therefore, we aim to implement a systematic review by synthesizing clinical experimental data combined with mass vaccination data and conducting a synthesis to evaluate the safety and efficacy of COVID-19 vaccines. Compared with other vaccines, adverse reactions after vaccination with inactivated vaccines are relatively low. The efficacy of inactivated vaccines is approximately 60%, adenovirus-vectored vaccines are 65%, and mRNA vaccines are 90%, which are always efficient against asymptomatic severe acute respiratory syndrome coronavirus 2 (SARS-CoV-2) infection, symptomatic COVID-19, COVID-19 hospitalization, severe or critical hospitalization, and death. RNA-based vaccines have a number of advantages and are one of the most promising vaccines identified to date and are particularly important during a pandemic. However, further improvements are required. In time, all the antibody levels weaken gradually, so a booster dose is needed to maintain immunity. Compared with homologous prime-boost immunization, heterologous prime-boost immunization prompts more robust humoral and cellular immune responses.

## 1. Introduction

After the initial outbreak in Wuhan, China, coronavirus disease 2019 (COVID-19) has now spread to more than 200 countries and territories [1]. The worldwide pandemic has imposed a challenge to human health, a significant test of humans facing public health emergencies. Due to ineffective treatments against severe acute respiratory syndrome coronavirus 2 (SARS-CoV-2), vaccination has become a primary strategy to control the COVID-19 pandemic. In the long term, the establishment of herd immunity by increasing population immunity above a threshold is extremely critical to viral eradication, which not only reduces the spread of the virus from person to person but also indirectly protects unvaccinated, high-risk individuals, including infants, pregnant or breastfeeding women, patients with cancer, immunocompromised people, and so on [2,3]. This can only be done by expanding the vaccinated population to achieve the SARS-CoV-2 herd immunity threshold as fast as possible for specific vaccines. Studies have suggested that the herd immunity threshold for SARS-CoV-2 is approximately 60–70% [4,5]. Therefore, wars against SARS-CoV-2 cannot be comprehensively won without vaccines.

The differences in technical principles and production routes of COVID-19 vaccines will necessarily lead to differences in safety and efficacy [6,7]. Therefore, we aim to implement a systematic review by synthesizing clinical experimental data combined with mass vaccination data and conducting a synthesis to evaluate the safety and efficacy of COVID-19 vaccines.

## 2. Concept of Vaccine Design and Mechanism of Action

SARS-CoV-2, the pathogen causing COVID-19, is a positive-stranded enveloped RNA virus containing four main structural proteins, including the spike protein (S), an envelope protein (E), membrane protein (M), and nucleocapsid protein (N) [8,9]. Among them, the S protein, primarily the receptor-binding domain (RBD) on the S1 subunit, mediates cell membrane fusion, facilitates endocytosis, and initiates intracellular signaling related to viral replication by binding to the cell surface receptor angiotensin-converting enzyme 2 (ACE2) [10,11]. Thus, the S protein is the primary target for vaccine design [12]. The role of vaccines is to artificially activate a beneficial immune response by inducing antibody and memory T-like cells. The immune response occurs rapidly upon viral invasion, leading to rapid anamnestic antibody and T cell responses. Circulating antibodies and memory cell recall responses can immediately eliminate viruses and limit virus dissemination [13].

## 3. Types and Differences of Vaccines

A vaccine from research to marketing ultimately typically requires 5 to 10 years. However, ongoing outbreaks and strong support from drug regulatory authorities greatly accelerate the process of vaccine development. At present, multiple COVID-19 vaccines have been included in the World Health Organization (WHO) emergency use list or have been granted emergency use authorization among different countries, including inactivated vaccines and adenovirus-vectored vaccines, and nucleic acid vaccines [14,15,16,17,18,19]. The vaccines were all given intramuscularly in the deltoid. More details are shown in Table 1.

**Figure 1:**
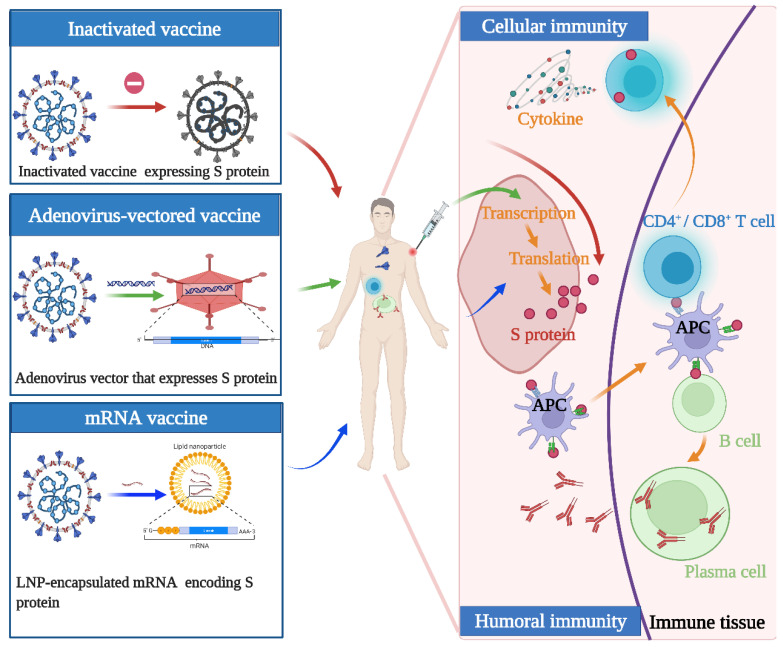
Illustration of the design and operation principles for different COVID-19 vaccines. Inactivated vaccines are inactivated but previously had virulent microorganisms that have been destroyed with chemicals, heat, or radiation. Adenovirus-vectored vaccines are recombinant vaccines formulated by combining the replication-deficient adenovirus vector and the target DNA. mRNA vaccines are a novel type of vaccine that works by injecting genetically engineered vectors containing RNA sequences encoding specific antigens. They function by activating cellular and humoral immunity to varying degrees.

Although the above vaccines fulfill the WHO criteria, the fundamental principles and production routes have varied. An illustration of the design principles for different COVID-19 vaccines is presented in Figure 1. Meanwhile, each of them has strengths and weaknesses. Inactivated viral vaccines are a mature technology that has been successfully used in immunization programs for decades, containing inactivated but previously virulent microorganisms that have been destroyed with chemicals, heat, or radiation [20]. In the absence of detailed information about pathogens, inactivated vaccines are the only available vaccine against pandemics [21]. However, inactivated vaccines frequently need to be given in multiple doses, and often a booster dose is necessary to maintain immunity. Examples include BBIBP-CorV (Sinopharm) and CoronaVac (Sinovac). Adenovirus-vectored vaccines are recombinant vaccines formulated by combining the replication-deficient adenovirus vector and the target DNA, such as Convidecia containing replication-defective adenovirus type-5 (Ad5) vectors and the full-length spike gene [22,23]. Therefore, immunized participants can produce S protein and thus, cause a protective immunological response. However, neutralizing antibodies against adenovirus are prevalent in the general population and are likely to weaken the protective efficacy of vaccines [24,25]. Nucleic acid (DNA and RNA) vaccines are novel types of vaccines that work by injecting genetically engineered vectors containing DNA/RNA sequences encoding specific antigens [26]. Nucleic acid vaccines have theoretical advantages over conventional vaccines, which can induce a variety of immune response types at the same time by artificial sequence design [27,28]. Compared with DNA vaccines, mRNA vaccines are safer and more efficient because they avoid the risk of integration with the host cell genome and can produce pure viral protein [29,30,31]. However, mRNA is destabilized and susceptible to degradation, which requires harsh preservation conditions, such as ultralow temperatures. Moreover, none of the mRNA vaccines have been licensed before, and experience in mass production is also scarce. Some mRNA vaccines among the COVID-19 vaccines have received emergency use authorization, such as BNT162b2 and mRNA-1273 developed by Pfizer/BioNTech and Moderna, respectively.

## 4. Safety

Safety is the first consideration of widespread vaccination, mainly assessed by adverse reactions (including local and systemic adverse reactions) and adverse event monitoring [32]. The vaccine recipients were monitored for 30 min postvaccination for any immediate adverse reactions. The most solicited common local adverse reactions are pain, redness, and swelling at the injection site, whereas the common systemic adverse reactions are fatigue, headache, fever, myalgia, diarrhea, nausea, cough, hypersensitivity, decreased appetite, and so on. Serious adverse events are rare and typically involve allergic reactions that can cause difficulty breathing [33]. Safety protection requirements are very demanding regarding the research and development of inactivated vaccines. Inactivated whole viruses can access the body directly and easily induce antibody-dependent enhancement (ADE) [34], a phenomenon in which binding a virus to suboptimal antibodies enhances its entry into host cells and replication, resulting in increased virus infectivity and virulence [35]. Since body immune mechanisms and genes encoding S structural proteins do not contain virus components, the safety of recombinant adenovirus-vectored vaccines should be good since being unable to self-replicate adenovirus can be eliminated. However, the clinical experiments of AstraZeneca and Johnson & Johnson on the recombinant adenovirus-vectored vaccine were paused because of the occurrence of thrombotic thrombocytopenia [36,37], which has raised questions about the safety of the adenovirus-vectored vaccine. Theoretically, nucleic acid vaccines, especially mRNA vaccines, are considered the safest vaccines without an infection risk because they do not contain any virus components and do not integrate with the host cell genome. However, anaphylactic reactions have been reported more after the administration of BNT162b2 [38]. The safe characteristic features of each representative vaccine will be discussed in detail as follows.

### 4.1. Safety of Inactivated Vaccine CoronaVac

Several manufacturers are developing inactivated SARS-CoV-2 vaccines against COVID-19. For example, CoronaVac was created from African green monkey kidney cells (Vero cells) after inoculation with SARS-CoV-2 (CZ02 strain) and developed by Sinovac [39]. Phase I/II; trials showed that the vaccine doses investigated (3 µg or 6 µg) had similar safety and immunogenicity profiles [40,41]. After carefully considering production capacity, 3 μg of inactivated SARS-CoV-2 virus in 0.5 mL of aluminum hydroxide diluent per dose with a two-dose immunization schedule was supported for clinical trial studies. CoronaVac has been granted emergency authorization in more than 32 countries or jurisdictions.

In a phase I/II; clinical trial of Chinese populations (including children and adolescents aged 3–17 years [42], adults aged 18–59 years [41], and adults aged 60 years and older [40]), the overall incidence of adverse reactions was 19–33% in the vaccine group and 18–22% in the placebo group across all age groups, with no significant difference between them. There were no significant differences in the incidence of local adverse reactions in all age groups except for injection-site pain among the population aged 3–59 years, which was also the most common local adverse reaction, reported by 10–21% of vaccine recipients and 2–10% of placebo recipients in all age groups. There were no significant differences in the incidence of systemic adverse reactions among all age groups. The most common systemic reaction was fever, reported by 3–5% of vaccine recipients and 1–4% of placebo recipients. No vaccine-related serious adverse events were reported after vaccination.

The phase III clinical trial in a Turkish population aged 18–59 years demonstrated that the frequencies of total adverse events were 18.9% in the vaccine group and 16.9% in the placebo group (*p* = 0.0108) with no fatalities or grade 4 adverse events [43]. Injection-site pain was the most common and the only local adverse reaction, with statistically significant differences (2.4% in the vaccine group and 1.1% in the placebo group, *p* < 0.0001). The frequency of systemic adverse reactions was significantly higher in the vaccine group (17.7%) than in the placebo group (16.0%, *p* = 0.0263), and specific symptoms with significant differences included fatigue, myalgia, and nausea (8.2%, 4.0%, 0.7% in the vaccine group and 7.0%, 3.0%, 0.2% in the placebo group, respectively, *p* < 0.05). One serious adverse event (grade 3 systemic allergic reaction) was reported to have a causal relationship with the vaccine, which occurred more than 24 h after the first dose of vaccine and resolved uneventfully in the following 24 h.

The phase III clinical trial in an Indonesian population aged 18–59 years showed that most adverse events were mild both in the vaccine (47.9%) and placebo (42.9%) groups [44]. The most common local adverse reaction and the most common systemic event were pain and myalgia, respectively (30.5% and 19.9% in the vaccine group vs. 30.1% and 9.0% in the placebo group). There were nine serious adverse events (SAEs) in all subjects including five not related to vaccines, one very unlikely, and three less likely to be related to vaccine products. Mass vaccination campaigns in Chile, including approximately 10.2 million persons, demonstrated fewer adverse events [45]. Any pattern among these deaths could not suggest a security issue for CoronaVac.

In conclusion, CoronaVac was well tolerated in populations across all age groups among different countries. The most common local adverse reaction was pain, whereas the common systemic events were fatigue, headache, fever, and myalgia. Most adverse reactions were slight and disappeared within a short time. There was almost no vaccine-related serious adverse reactions observed after vaccination. ADE is a theoretical possibility with inactivated vaccines. Clinical trials in people thus far have not shown that participants who received the vaccine have a higher rate of severe illness than participants who did not receive the vaccine.

### 4.2. Safety of Adenoviral Vector Vaccine Convidecia

In recent years, recombinant adenovirus has been widely developed as a vaccine vector, which is also a crucial technical route for vaccines against COVID-19. Among them, Convidecia is the first vaccine against COVID-19 in a human trial and the only vaccine with two modes of administration, including intramuscular injection and inhalation. Convidecia was constructed by cloning an optimized full-length spike gene into an E1, and E3 deleted Ad5 vector [22]. The mass vaccination dose and immunization schedule (5 × 10^10^ viral particles per 0.5 mL, a single injection) chosen are mainly based on the data from phase I/II; clinical trials.

In phase I/II; clinical trials of healthy Chinese individuals from Wuhan (phase I; for adults aged 18–60 years; phase II; for participants aged ≥18 years, 13.0% for ≥55 years), 76.0% of recipients in the vaccine group and 48.0% in the placebo group experienced at least one or more adverse events within 28 days after vaccination. A total of 74.0% of participants in the vaccine group reported at least one solicited adverse reaction within 14 days after vaccination, which was significantly higher than the 37.0% in the placebo group [46,47,48]. The most common local solicited adverse reaction was pain, reported by 56.0% vaccine recipients and 9.0% placebo recipients. The most common systemic solicited reactions in the vaccine and placebo groups were fatigue (34.0% vs. 17.0%), fever (16.0% vs. 10.0%) and headache (28.0% vs. 13.0%), respectively. All of the above metrics in the vaccine group were significantly higher than those in the placebo group. Unsolicited adverse reactions within 14 days postvaccination showed no difference across the groups. No vaccine-related serious adverse events were documented within 28 days.

Phase III clinical trials in Pakistan, Mexico, Russia, Chile, and Argentina (78 clinical research centers in total) were completed. Adverse reactions were monitored over 52 weeks. A total of 61.3% of vaccine recipients and 20.0% of placebo recipients reported an injection-site adverse event (*p* < 0.0001), of which pain was the most frequent, reported by 59.0% vaccine recipients and 19.0% placebo recipients. A total of 63.5% of vaccine recipients and 46.4% of placebo recipients reported a solicited systemic adverse event (*p* < 0.0001), of which headache was the most common (44.0% of vaccine recipients and 30.6% of placebo recipients; *p* < 0.0001) [49].

A phase I clinical trial of the aerosolized adenovirus type-5 vector-based COVID-19 vaccine (Ad5-nCoV) is just being finished. Safety evaluation revealed that no significant difference in the incidence of any solicited adverse events was found between the two vaccination routes within 7 days after the first vaccination or booster vaccination. However, injection-site pain was avoided. No vaccine-related serious adverse events were noted within 56 days after the first vaccination.

Although a significantly higher proportion of participants in the vaccine group reported adverse reactions such as fever, fatigue, and injection site pain than participants in the placebo group, adverse reactions were generally mild and resolved in no more than 48 h. The experimental aerosolized COVID-19 vaccine with promising potential has a good safety profile.

### 4.3. Safety of the mRNA Vaccine BNT162b2

Nucleic acid vaccines have emerged as ideal methods for rapid vaccine design, including DNA and mRNA vaccines. Among them, BNT162b2, the first vaccine against COVID-19 to be approved by the WHO for emergency use, is a lipid nanoparticle-formulated, nucleoside-modified RNA vaccine that encodes a prefusion stabilized, membrane-anchored SARS-CoV-2 full-length S protein [10,50]. After injection, the lipid nanoparticle-formulated mRNA vaccine is taken up by the cells, and the RNA is released into the cytosol, where it is translated into the S protein.

The multinational clinical trial of BNT162b2 in a population aged 16 years of age or older demonstrated that BNT162b2 recipients reported more local reactions than placebo recipients. The most commonly reported local adverse response in participants was pain, which was reported more frequently in younger participants (83.0% vs. 78.0% after the first and second dose) than participants older than 55 years of age (71.0% vs. 66.0% after the first and second dose) [51]. Injection-site redness and swelling were reported by a noticeably lower percentage of participants. Most local reactions were mild to moderate in severity and usually resolved within 48 h.

Systemic events were reported more often by younger vaccine recipients than older vaccine recipients in the reactogenicity subset and more often after dose 2 than dose 1. Fatigue and headache were the most commonly reported systemic events, which were higher in vaccine recipients (59.0% and 52.0%, among 16–55 years; 51.0% and 39.0%, among ≥55 years) than in placebo recipients (23.0% and 24.0%, among 16–55 years; 17.0% and 14.0%, among ≥55 years). The frequency of any severe systemic event after the first dose was 0.9% or less. Fever (≥38 °C) was reported after the second dose by 16.0% of younger vaccine recipients and 11.0% of older recipients. Only 0.2% of vaccine recipients and 0.1% of placebo recipients reported a fever (38.9–40 °C) after the first dose, compared with 0.8% and 0.1% after the second dose, respectively. Two participants in the vaccine and placebo groups reported temperatures above 40.0 °C.

Adverse events or related adverse events were reported more frequently in BNT162b2 recipients (27.0% and 21.0%) than in placebo recipients (12.0% and 5.0%) [51]. Sixty-four vaccine recipients (0.3%) and six placebo recipients (<0.1%) reported lymphadenopathy. The CDC identified and submitted the Vaccine Adverse Event Reporting System (VAERS), which estimated that the rate of anaphylaxis was 11.1 cases per million doses administered [38]. Four serious adverse events related to vaccination were reported among BNT162b2 recipients, including shoulder injury, paroxysmal ventricular arrhythmia, right axillary lymphadenopathy, and right leg paresthesia [51]. Six recipients died (four from the placebo group and two from the vaccine group). However, the findings showed that deaths were unrelated to vaccines or placebo. Multiple articles recently reported that many healthy young individuals were definitively diagnosed with myocarditis after receiving the second dose of the mRNA vaccine [52,53]. Cardiac MRI demonstrated that individuals with vaccine-associated myocarditis have a similar pattern of myocardial injury [54]. Further investigations are necessary to better understand the causal association. Meanwhile, some sporadic articles also reported that vaccinated individuals developed a chronic obstructive pulmonary disease and acute exacerbation of idiopathic pulmonary fibrosis [55,56].

Adverse reactions were reported more often by vaccine recipients than placebo recipients, more often by younger vaccine recipients (16–55 years) than by older vaccine recipients (≥55 years), and more often after dose 2 than dose 1. Until now, several serious adverse reactions have been reported in individuals after receiving the mRNA vaccine, including anaphylactic reactions, lymphadenopathy, myocarditis, and pulmonary fibrosis, but the specific causal relationship needs to be proven in further studies.

### 4.4. Comprehensive Safety Evaluation

**Figure 2:**
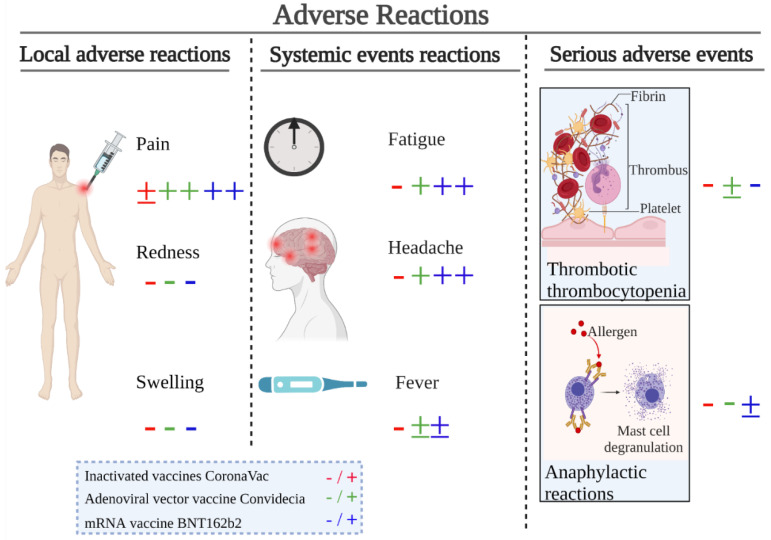
Illustration of common adverse reactions induced by COVID-19 vaccines. The most common local adverse reactions are pain, redness, and swelling at the injection site, whereas the common systemic adverse reactions are fatigue, headache, and fever. Serious adverse events are very rare and typically involve thrombotic thrombocytopenia and anaphylactic reactions. The incidence of each adverse reaction is denoted by different colors and symbols, with red indicating CoronaVac, green indicating Convidecia, blue indicating BNT162b2, and ‘−’ indicating incidence is less than 10%, ‘±’ indicating incidence is 10–20%, ‘+’ indicating incidence is 20–50%, and ‘++’ indicating incidence is greater than 50%.

Each vaccine must undergo rigorous clinical trials to ensure safety before approval. However, no vaccine can be 100% safe for everyone because each person’s body can react differently [57]. Minor side effects are relatively common, while serious side effects should be infrequent and occur in approximately 1 out of every 100,000 vaccinations. Injection-site pain was the most commonly reported local adverse reaction in the above vaccines through intramuscular injection, while fatigue and headache were the most frequently reported systemic adverse reactions. Several recent clinical trials are currently in the development pipeline to deliver vaccines via mucosal surfaces to be taken up by the common mucosal immune system, thus avoiding local adverse reactions [58]. Most adverse reactions were mild in severity, and participants recovered within a short period. The comparability of adverse reactions between vaccines may be limited due to differences in the definition of the threshold for the same behavior. However, compared with their respective controls, the available results could explain some problems to a certain extent. Figure 2 summarizes the crude effects of this comparison. There were very few differences in the incidence of adverse reactions across all age groups between vaccine recipients and placebo recipients for the inactivated vaccine CoronaVac. For the adenovirus-vectored vaccine Convidecia and mRNA vaccine BNT162b2, vaccine recipients reported more adverse reactions than placebo recipients. Anaphylactic reactions and lymphadenopathy have been reported more after the administration of BNT162b2 [38]. Vaccination with the adenovirus-vectored vaccine (ChAdOx1 nCov-19) can result in the rare development of immune thrombotic thrombocytopenia [36]. Compared with other COVID-19 vaccine candidates, adverse reactions after vaccination with inactivated vaccines were relatively low. Adenovirus-vectored vaccines and mRNA vaccines have a number of advantages; however, further improvements are required.

The incidence of severe adverse reactions, such as allergic reactions, is not merely correlated with the vaccine itself but is also related to the ingredients of vaccines and the physical characteristics of recipients. For example, polyethylene glycol (PEG), used in mRNA vaccines as a lipid carrier, was associated with an increasing number of PEG-associated anaphylaxis events [59]. A total of 81.0% of patients with anaphylaxis have a documented history of allergies or allergic reactions, and 33% have experienced an episode of anaphylaxis in the past [38]. In the case of any severe adverse reactions, careful understanding of the medical history and allergic diseases was necessary to avoid adverse reactions before vaccination. The areas of personnel scheduling, material allocation, emergency planning, and workflow should be carefully planned before vaccination. Postvaccination observation periods for 30 min are of great necessity and ensure sufficient quantities of epinephrine [58].

## 5. Effectiveness and Immunogenicity

Effectiveness is a better marker than all vaccine quality variables except for safety. Vaccine efficacy is the most important and intuitive evaluation metric, assessed by comparing the percentage of reduction in disease incidence in a vaccinated versus unvaccinated population or vaccine-specific antibody production. High immunogenicity is pivotal for high vaccine efficacy and represents a fundamental challenge for vaccine development, closely related to strong humoral and cellular immune responses in vaccine recipients [60,61,62]. The geometric mean titers (GMTs) of specific antibody responses to the RBD and neutralizing antibody amounts against live SARS-CoV-2 and seroconversion (a positive antibody response is at least a fourfold increase in postvaccination titer from baseline) were measured as humoral immunogenicity endpoints. RBD antibody closed the receptor-binding domain on the S1 subunit of SARS-CoV-2 and thereby prevented membrane fusion and endocytosis, which could lead to viral replication. Neutralizing antibodies prevented the virus from interacting with its host cells by neutralizing the biological effects of the antigen without a need for immune cells. The higher the GMTs of the antibody is, the better the humoral immune effect. Specific T cell response quantification with an interferon (IFN)γ enzyme-linked immunospot (ELISpot) assay and positive T cell responses according to the secretion of cytokines, such as IFNγ, interleukin-2 (IL-2), and tumor necrosis factor α (TNF-α), are detected as endpoints for cellular immune responses. It is known that many factors affect immunogenicity, including the technical principles of vaccines and the personal baseline characteristics of recipients. For example, compared with recipients with high pre-existing anti-Ad5 immunity, recipients with low pre-existing anti-Ad5 immunity had more elevated RBD-specific antibodies and neutralizing antibodies [46].

### 5.1. Effectiveness and Immunogenicity of the Inactivated Vaccine CoronaVac

The phase III study of CoronaVac has been conducted in multiple countries, including Brazil, Turkey, Indonesia, and Chile. Based on the interim result from health care workers with a vaccination schedule (day 0/14) in Brazil (5.1% for ≥60 years; 36.0% for males), the overall vaccine efficacy against any COVID-19 was 50.7% (50.7% for 18–59 years; 51.1% for ≥60 years), while the vaccine efficacy against hospitalization and severe COVID-19 was 100.0% [14]. Vaccine efficacy was similar among participants with any comorbidity (48.9%), including hypertension 100.0%, obesity 74.9%, and type 2 diabetes mellitus 48.6%. Based on the interim result in a population aged 18–59 years with a vaccination schedule (day 0/14), vaccine efficacy in Turkey and Indonesia was 83.5% and 65.3% [43,44], respectively. Mass vaccination campaigns in a population aged 16 years of age or older in Chile (26.2% for ≥60 years) showed that the vaccine effectiveness was 65.9% (66.6% for ≥60 years) for the prevention of COVID-19, 87.5% for the prevention of hospitalization, 90.3% for the prevention of ICU admission, and 86.3% for the prevention of COVID-19-related death [45].

Overall, the findings suggested that CoronaVac was highly effective in protecting against symptomatic SARS-CoV-2 infection, severe COVID-19, and death. The effectiveness results in persons aged 18–59 years were also consistent with those for persons 60 years of age or older. Vaccine efficacy was similar among participants with any comorbidity. The variability of vaccine efficacy between the countries could be of great relevance for variance in study characteristics such as population, sample size, detection reagents, and force of infection. Brazil has the lowest vaccine efficacy, which may be related to the high-risk population, particularly health care workers.

The humoral immunity results from Indonesia showed that the GMTs and seroconversion rates of neutralizing antibodies were significantly different between the vaccine group and placebo group at day 14 post-vaccination (15.8, 87.2% vs. 2.0, 0.0%). The GMTs and seroconversion rates of RBD-specific IgG also varied significantly between the vaccine and placebo groups at day 14 post-vaccination (5181.2, 97.5% vs. 223.6, 0.8%) [44]. Similar to phase I/II trials [41], the GMTs and seroconversion rates of neutralizing antibodies in the day 0/14 cohort showed significant differences between the vaccine group and placebo group at day 14 post-vaccination (27.6, 92.0% vs. 0.0, 3.0%) and at day 28 post-vaccination (23.8, 94.0% vs. 0.0, 0.0%), which also varied significantly at day 28 post-vaccination (44.1, 97.0% vs. 0.0, 0.0%) in the day 0/28 cohort. The GMTs and seroconversion rates of RBD-specific IgG in the day 0/14 cohort showed significant differences between the 3 µg group and the placebo group at day 14 post-vaccination (1094.3, 97.0% vs. 81.0, 0.0%) and at day 28 post-vaccination (1053.7, 97.0% vs. 80.0, 0.0%). There were marked differences between the 3 µg group and the placebo group at day 28 post-vaccination (1783.6, 99% vs. 87.9, 7%) in the day 0/28 cohort. The phase I/II trial results reported here also demonstrated that the humoral immune response in the (day 0/28) vaccination schedule was larger than that in the (day 0/14) vaccination schedule.

Humoral immunity results from Turkey indicated that 89.7% of vaccines produced seropositive RBD-specific antibodies, and 92.0% of seropositive vaccines also yielded protective neutralizing antibodies at least 14 days after the second vaccine. Seropositivity decreased significantly with increasing age in women and men aged 18–59 [43], consistent with phase I/II trials in China. In the above phase I/II trials, the GMTs and seroconversion rates of neutralizing antibodies at day 28 post-vaccination showed significant differences between the 3 µg group and placebo group in phase 1 (54.9, 100.0% vs. 0.0, 0.0%) and in phase 2 (42.2, 98.0% vs. 0.0, 0.0%) among the population over the age of 60 years [40], which also varies significantly between them in phase 1 (117.4, 100.0% vs. 0.0, 0.0%) and in phase 2 (142.2, 100.0% vs. 0.0, 0.0%) among the population aged 3–17 years [42].

The immunogenicity analysis demonstrated that CoronaVac had good consistency between each batch and appeared to induce a strong humoral immune response in all phase I/II/III trials. Compared with a 0/14 day emergency schedule, a 0/28 day routine induced more robust humoral immune responses. Compared with the population over the age of 17 years, CoronaVac induced more robust humoral immune responses among the population aged 3–17 years.

### 5.2. Effectiveness and Immunogenicity of Adenovirus-Vectored Vaccine Convidecia

The interim and final analysis data showed that Convidecia had an overall efficacy of 65.3% or 57.5% at preventing all symptomatic COVID-19 disease 28 days after single-dose vaccination and 68.8% or 63.7% 14 days after single-dose vaccination. Convidecia has an efficacy of 90.1% or 91.7% at preventing severe disease 28 days postvaccination and 95.5% or 96.0% 14 days postvaccination [49].

The humoral immunity results from the phase Ⅱ clinical trial showed that RBD-specific antibody GMTs and seroconversion rates were 571.0 and 97.0%, respectively, at day 28 post-vaccination. In contrast, no antibody increase from baseline was 20.7 in the placebo group. The GMTs and seroconversion rates of neutralizing antibodies were 18.3 and 47.0%, respectively, at day 28 post-vaccination, and no antibody increase from baseline was observed at 4.1 in the placebo group. Vaccinees with low pre-existing anti-Ad5 immunity had approximately two times higher expression of RBD-specific antibody and neutralizing antibody than those with high pre-existing anti-Ad5 immunity. Increasing age was inversely related to the production of RBD-specific antibodies and neutralizing antibody responses. Male and female participants presented similar levels of RBD-specific antibodies and neutralizing antibodies post-vaccination.

T cell response results showed that 88.0% of participants in the vaccine groups were positive for SARS-CoV-2 spike glycoprotein-specific IFNγ responses, whereas there were no positive responses in the placebo group. Both CD4+ T cells and CD8+ T cells were activated in vaccine recipients. T cell responses were observed in participants with high and low pre-existing neutralizing antibodies on day 28. However, a pre-existing Ad5 neutralizing antibody harmed the pattern of T cell responses. The sex and age of the participants did not differ in their IFNγ T cell responses post-vaccination.

The immunogenicity results of aerosolized Ad5-nCoV showed [58] that at day 28 after the last vaccination, the geometric mean concentrations of IgG, IgA, and GMTs of neutralizing antibody were 261 EU/mL, 312 EU/mL, and 107 in participants who received two high aerosolized doses (2 × 10^10^ viral particles); 289 EU/mL, 297 EU/mL, and 105 in participants who received two low aerosolized doses (1 × 10^10^ viral particles); 2013 EU/mL, 777 EU/mL, and 396 in participants who received an initial intramuscular (5 × 10^10^ viral particles) vaccine followed by an aerosolized booster (2 × 10^10^ viral particles); 915 EU/mL, 425 EU/mL, and 95 in participants who received one intramuscular dose (5 × 10^10^ viral particles); and 1190 EU/mL, 521 EU/mL, 425 EU/mL, and 95 in participants who received one intramuscular dose (10^11^ viral particles). The T cell response results showed that one dose of aerosolized Ad5-nCoV induced broad T cell responses, similar to the phenotype observed with intramuscular Ad5-nCoV. CD4+ T cells predominantly secrete T helper-1 cytokines (IFN-γ and IL-2) rather than T helper-2 cytokines (IL-4 and IL-13). Pre-existing Ad5 neutralizing antibody significantly reduced the specific IFN-γ response. The mixed group received an initial intramuscular vaccine and an aerosolized booster that exhibited the best immune effects.

In summary, adenovirus-vectored vaccines could induce robust humoral responses, including RBD-binding IgG, IgA, and SARS-CoV-2 neutralizing antibodies and cellular immune responses after only a single immunization. Similar to some clinical trials [63,64], pre-existing immunity against adenovirus vectors affected the potency of an adenovirus-based vaccine by both humoral immunity and cellular immunity. Mixed vaccination strategies of intramuscular and aerosolized boosters could result in better efficacy and effectiveness, but further studies are required to elucidate the details.

### 5.3. Effectiveness and Immunogenicity of the mRNA Vaccine BNT162b2

This nationwide observational study in Israel showed that vaccine effectiveness estimates against all SARS-CoV-2 outcomes were slightly higher at 14 days or longer than at 7 days or longer after the second dose. Vaccine effectiveness was 95.3% and 96.5% against SARS-CoV-2 infection, 91.5% and 93.8% against asymptomatic SARS-CoV-2 infection, 97.0% and 97.7% against symptomatic COVID-19, 97.2% and 98.0% against COVID-19 hospitalization, 97.5% and 98.4% against severe or critical hospitalization, and 96.7% and 98.1% against death at 7 days or longer and at 14 days and long after the second dose, respectively [65]. These findings are consistent with phase I/II trials that indicated that BNT162b2 was 95.0% effective in preventing COVID-19 [51]. Similar vaccine efficacy (generally 90 to 100%) was observed across subgroups defined by age, sex, race, ethnicity, baseline body mass index, and the presence of coexisting conditions. Between the first and second doses, vaccine efficacy was 52.0% during this interval, indicating early protection by the vaccine, starting as soon as 12 days after the first dose. Compared with individuals with intact immune systems, vaccinated patients with hematological neoplasms were more susceptible to COVID-19, suggesting that vaccine effectiveness in patients with hematological neoplasms was decreased [66].

The humoral immunity results from the phase I clinical trial showed that the GMTs of neutralizing antibody elicited by BNT162b2 peaked one week after the second vaccination and began decaying one week later. RBD-specific antibodies were 9136.0 and 8147.0 U/mL among participants 18 to 55 years of age, or 7985.0 and 6014.0 U/mL among those 65 to 85 years of age 7 or at day 14 post-vaccination, whereas no antibody increase from baseline was 0.9 U/mL in the placebo group. The 50% neutralization titers were 361.0 and 163.0 among participants 18 to 55 years of age and 149.0 and 206.0 among those 65 to 85 years of age at day 7 or at day 14 post-vaccination, respectively, whereas no titer increase from baseline was 10.0 in the placebo group. The 50% neutralizing GMTs at day 7 post-vaccination or day 14 post-vaccination ranged from 1.7 to 4.6 times the GMT of the convalescent serum panel among participants 18 to 55 years of age and from 1.1 to 2.2 times the GMT of the convalescent serum panel among those 65 to 85 years of age [67]. BNT162b2 elicited generally lower antigen-binding IgG and virus-neutralizing responses in participants 65 to 85 years of age than in those 18 to 55 years of age.

Cell response results showed that a single dose of BNT162b2 elicited weak neutralizing activity elicited in SARS-CoV-2-naïve individuals but strong anti-RBD and anti-S antibodies with Fc-mediated effector functions and cellular CD4+/CD8+ T cell responses [68]. Strong correlations between T helper cells and humoral responses showed that CD4+ T cell responses facilitated the generation of specific humoral responses against SARS-CoV-2 after a single dose of BNT162b2. Compared to naïve people, participants with a history of infection elicited more robust and functionally skewed responses. SARS-CoV-2-naïve individuals with BNT162b2 stimulated specific humoral and T cell responses to levels similar to those presented in infected individuals approximately nine months ago.

An exploratory analysis reported that the mRNA vaccine was immunogenic in pregnant women, including strong antibody responses and T-cell responses, and the induced antibodies could be transported to infants by cord blood and breast milk. The GMTs of neutralizing antibodies in nonpregnant, pregnant, and lactating women were lower by 3.5-fold for the B.1.1.7 variant and 6-fold lower for the B.1.351 variant than for the wild-type variant [69].

Together, the BNT162b2 vaccine could induce robust humoral responses, including RBD-binding IgM IgG, IgA, and SARS-CoV-2 neutralizing antibodies and cellular immune responses after only a single immunization. Moreover, there was a strong correlation between T helper cells and humoral responses.

### 5.4. Comprehensive Effectiveness Evaluation

**Figure 3:**
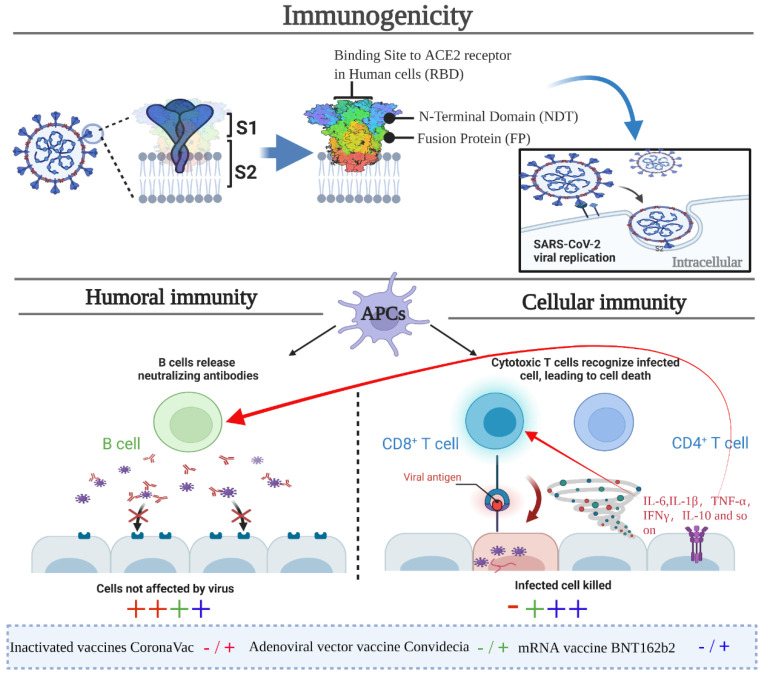
Illustration of immunogenicity induced by COVID-19 vaccines. Both neutralizing antibody and T cell responses are important in eradicating the virus and controlling COVID-19 development. Neutralizing antibodies exert effects by neutralizing free viruses. T cell responses are essential for directly killing virus-infected cells. In addition, CD4+ cell responses are critical for the cytotoxic T cell response and for antibody production in B cells. The intensity of immunogenicity is denoted by different colors and symbols, with red indicating CoronaVac, green indicating Convidecia, blue indicating BNT162b2, ‘−’ indicating the unreported intensity, ‘+’ indicating the intensity is moderate, and ‘++’ indicating the intensity is strong.

According to WHO target product profiles for COVID-19 vaccines [70], the characteristics required for emergency use during an outbreak included efficacy of at least 50%, maximum of two-dose regimen, suitability for use in older adults, and protection for at least 6 months. Vaccines with emergency use authorization all achieved WHO criteria. The efficacy of inactivated vaccines was approximately 60%, adenovirus-vectored vaccines were 65%, and mRNA vaccines were 90%, which were always efficient against asymptomatic SARS-CoV-2 infection, symptomatic COVID-19, COVID-19 hospitalization, severe or critical hospitalization, and death. It could be summarized from the above COVID-19 vaccines that similar overall vaccine efficacy was observed across subgroups defined by age, but increasing age was found to be negatively correlated with the production of RBD-specific antibodies and neutralizing antibody responses. Figure 3 summarizes the immunogenicity results of COVID-19 vaccines. Neutralizing antibody and T cell responses are essential in eradicating the virus and controlling COVID-19 progression. Neutralizing antibodies exert effects by neutralizing free viruses. T cell responses are crucial in killing virus-infected cells. CD4+ cells play important roles in B cell maturation and the development of high-affinity antibodies in the germinal center of secondary lymphoid organs. Both Convidecia and BNT162b2 induced robust humoral and T cell immune responses after vaccination. However, reports about CoronaVac-induced T cell responses are currently lacking. Compared with conventional immunity, aerosolized immunity has some unique strengths in triggering mucosal immunity and in preventing invading pathogens.

Notably, vaccine effectiveness was gradually weakened over time. Robust evidence demonstrated that the protective efficacy against SARS-CoV-2 infection decreased by 21.0% over six months from full vaccination across all ages and for different vaccine types, including mRNA vaccines and adenovirus-vectored vaccines [71]. However, vaccine efficacy against severe disease remained greater than 70% over time. A subsequent study also confirmed the similar conclusion that the protection efficacy waned to negligible levels within seven months for BNT162b2 and 4 months for ChAdOx1 nCoV-19 [72]. The decreased effectiveness was related not only to waning immunity but also to the emergence of new variants. The emergence of new variants appeared to accelerate the waning of vaccine protection. Compared with the earlier delta variant, the decline in protection efficacy was more distinctive for omicron even in the first month after a booster dose [73]. SARS-CoV-2 was unlikely to be eliminated within a short time. Hence, vaccines with broad and durable protection are urgently needed.

## 6. Prime-Boost Strategies

It should be noted that the antibody level gradually decreased over time [74]. The third or fourth dose of vaccine could be necessary to sustain and prolong the duration of protection in populations that have completed vaccination regimens. At present, multiple prime-boost strategies, including homologous and heterologous strategies, are being explored. Heterologous prime-boost immunization improved both humoral and cellular immune responses, confirmed first in a mouse model [75]. The GMTs of neutralizing antibody in recipients was 197.4 with 78-fold elevation after boosting with adenovirus-vectored Convidecia following two doses of inactivated vaccine CoronaVac at day 14 post-vaccination, while they were 33.6 with 15.2-fold elevation after boosting with the third dose of inactivated vaccine. The GMTs of neutralizing antibodies in recipients were 54.4 and 25.7-fold elevated after one dose of inactivated vaccine and one dose of adenovirus-vectored vaccines, respectively, while they were 12.8 and 6.2-fold elevated after two doses of inactivated vaccine, respectively. For the same vaccine, compared with two doses of aerosolized or one dose of intramuscular Convidecia, the mixed group received an initial intramuscular vaccine on day 0 followed by an aerosolized booster on day 28 and exhibited the best immune effects [58]. Studies have indicated that primary immunization provides limited protection against omicron-caused symptomatic disease after two doses of the ChAdOx1 nCoV-19 or BNT162b2 vaccine. Heterologous prime-boost immunization (a BNT162b2 or mRNA-1273 booster after either the ChAdOx1 nCoV-19 or BNT162b2 primary course) substantially increased protection against omicron and that protection also waned over time [73].

## 7. Conclusions

Compared with other vaccines, adverse reactions and immunogenicity after vaccination with the inactivated vaccine are all relatively low. mRNA vaccines with the highest effectiveness are one of the most promising vaccines identified to date. As long as viral genetic sequence information is known, RNA-based vaccines can be rapidly produced, particularly important during a pandemic. Taken together, there were sustained declines in SARS-CoV-2 incidence, suggesting that vaccination offers hope for eventual control of the COVID-19 outbreak. However, with new variants, especially those less susceptible to vaccines, progress toward herd immunity can be disrupted.

## Figures and Tables

**Table 1:** Several granted COVID-19 vaccines and details.

Vaccine Name	Technology	Developer/Company	Expiration Date	Immunization Protocol	Approved
CoronaVac	Inactivated vaccine	Sinovac Biotech Ltd. (Beijing, China)	2–8 °C for 24 months	2 doses (600SU/0.5 mL/dose), 2–4 weeks apart	WHO 2021.6.1
BBIBP-CorV	Inactivated vaccine	Sinopharm Beijing Institute of Biotechnology (Beijing, China)	2–8 °C for 24 months	2 doses (6.5U/0.5 mL/dose), 3–4 weeks apart	WHO 2021.5.7
Convidecia	Adenovirus vector vaccine	Cansino Biologics (Tianjin, China)	2–8 °C for 12 months	1 dose (5 × 10^10^ virus particles/0.5 mL)	China 2021.2. 25
AZD1222	Adenovirus vector vaccine	AstraZeneca (Cambridge, UK), Oxford University (Oxford, UK)	2–8 °C for 6 months	2 dose (5 × 10^10^ virus particles/0.5 mL), 4–12 weeks apart	WHO 2021.3.1
Ad26.COV2.S	Adenovirus vector vaccine	Johnson & Johnson (New Brunswick, NJ, USA)	2–8 °C for 3 months	1 dose (5 × 10^10^ virus particles/0.5 mL)	WHO 2021.3.17
Sputnik V	Adenovirus vector vaccine	Gamaleya Research Institute (Moscow, Russia)	−18 °C/2–8 °C	2 dose (10^11^ viral particles /0.5 mL/dose), 2–3 weeks apart	Multiple countries without WHO
BNT162b2	mRNA vaccine	Pfizer (New York, NY, USA)/BioNTech (Mainz, Germany)	Ultralow-temperature freezer for 6 months/−70 ± 10 °C for 10 days/2–8 °C for 5 days	2 doses (30 μg/0.3 mL/dose), 3 weeks apart	WHO 2021.1.14
mRNA-1273	mRNA vaccine	Moderna (Cambridge, MA, USA)	Between −25 °C and −15 °C for supply/2–8 °C for 30 days	2 doses (100 μg/0.5 mL/dose), 28 days apart	WHO 2021.2.3
NVX-CoV2373	Recombinant vaccine	Novavax and the Serum Institute of India (Pune, India)	2–8 °C for 9 months	2 doses (55 μg/0.5 mL/dose), 3–4 weeks apart	WHO 2021.12.20

## Data Availability

The data that support the findings of this study are available from the corresponding author upon reasonable request.

## References

[1] Zhu N., Zhang D., Wang W., Li X., Yang B., Song J., Zhao X., Huang B., Shi W., Lu R. (2020). A Novel Coronavirus from Patients with Pneumonia in China, 2019. N. Engl. J. Med..

[2] Okell L.C., Verity R., Watson O.J., Mishra S., Walker P., Whittaker C., Katzourakis A., Donnelly C.A., Riley S., Ghani A.C. (2020). Have deaths from COVID-19 in Europe plateaued due to herd immunity?. Lancet.

[3] Anderson R.M., Vegvari C., Truscott J., Collyer B.S. (2020). Challenges in creating herd immunity to SARS-CoV-2 infection by mass vaccination. Lancet.

[4] Angulo F.J., Finelli L., Swerdlow D.L. (2020). Reopening Society and the Need for Real-Time Assessment of COVID-19 at the Community Level. JAMA.

[5] Gomes M.G.M., Corder R.M., King J.G., Langwig K.E., Souto-Maior C., Carneiro J., Gonçalves G., Penha-Gonçalves C., Ferreira M.U., Aguas R. (2020). Individual variation in susceptibility or exposure to SARS-CoV-2 lowers the herd immunity threshold. medRxiv.

[6] Holvast A., Huckriede A., Wilschut J., Horst G., De Vries J.J., Benne C.A., Kallenberg C.G., Bijl M. (2006). Safety and efficacy of influenza vaccination in systemic lupus erythematosus patients with quiescent disease. Ann. Rheum. Dis..

[7] Zolla-Pazner S., Michael N.L., Kim J.H. (2021). A tale of four studies: HIV vaccine immunogenicity and efficacy in clinical trials. Lancet HIV.

[8] Kanimozhi G., Pradhapsingh B., Singh Pawar C., Khan H.A., Alrokayan S.H., Prasad N.R. (2021). SARS-CoV-2: Pathogenesis, Molecular Targets and Experimental Models. Front. Pharmacol..

[9] Prates E.T., Garvin M.R., Pavicic M., Jones P., Shah M., Demerdash O., Amos B.K., Geiger A., Jacobson D. (2021). Potential Pathogenicity Determinants Identified from Structural Proteomics of SARS-CoV and SARS-CoV-2. Mol. Biol. Evol..

[10] Wrapp D., Wang N., Corbett K.S., Goldsmith J.A., Hsieh C.L., Abiona O., Graham B.S., McLellan J.S. (2020). Cryo-EM structure of the 2019-nCoV spike in the prefusion conformation. Science.

[11] Zhang Z., Zhang Y., Liu K., Li Y., Lu Q., Wang Q., Zhang Y., Wang L., Liao H., Zheng A. (2021). The molecular basis for SARS-CoV-2 binding to dog ACE2. Nat. Commun..

[12] Dai L., Gao G.F. (2021). WHO Targets for Vaccines against COVID-19. Nat. Rev. Immunol..

[13] Plotkin S. (2014). History of vaccination. Proc. Natl. Acad. Sci. USA.

[14] World Health Organization Background Document on the Inactivated Vaccine Sinovac-CoronaVac against COVID-19. https://www.who.int/publications/i/item/WHO-2019-nCoV-vaccines-SAGE_recommendation-Sinovac-CoronaVac-background-2021.1.

[15] World Health Organization Background Document on the Inactivated COVID-19 Vaccine BIBP Developed by China National Biotec Group (CNBG), Sinopharm. https://www.who.int/publications/i/item/WHO-2019-nCoV-vaccines-SAGE_recommendation-BIBP-background-2021.1.

[16] World Health Organization Background Document on the AZD1222 Vaccine against COVID-19 Developed by Oxford University and AstraZeneca. https://www.who.int/publications/i/item/background-document-on-the-azd1222-vaccine-against-covid-19-developed-by-oxford-university-and-astrazeneca.

[17] World Health Organization Background Document on the mRNA-1273 Vaccine (Moderna) against COVID-19. https://www.who.int/publications/i/item/background-document-on-the-mrna-1273-vaccine-(moderna)-against-covid-19.

[18] World Health Organization Background Document on the mRNA Vaccine BNT162b2 (Pfizer-BioNTech) against COVID-19. https://www.who.int/publications/i/item/background-document-on-mrna-vaccine-bnt162b2-(pfizer-biontech)-against-covid-19.

[19] World Health Organization Background Document on the Janssen Ad26.COV2. S (COVID-19) Vaccine. https://www.who.int/publications/i/item/WHO-2019-nCoV-vaccines-SAGE-recommendation-Ad26.COV2.S-background-2021.1.

[20] Li J.X., Zhu F.C. (2021). Inactivated SARS-CoV-2 vaccine (BBV152)-induced protection against symptomatic COVID-19. Lancet.

[21] Chua B.Y., Wong C.Y., Mifsud E.J., Edenborough K.M., Sekiya T., Tan A.C., Mercuri F., Rockman S., Chen W., Turner S.J. (2015). Inactivated Influenza Vaccine That Provides Rapid, Innate-Immune-System-Mediated Protection and Subsequent Long-Term Adaptive Immunity. mBio.

[22] Wu S., Zhong G., Zhang J., Shuai L., Zhang Z., Wen Z., Wang B., Zhao Z., Song X., Chen Y. (2020). A single dose of an adenovirus-vectored vaccine provides protection against SARS-CoV-2 challenge. Nat. Commun..

[23] Roy C.J., Ault A., Sivasubramani S.K., Gorres J.P., Wei C.J., Andersen H., Gall J., Roederer M., Rao S.S. (2011). Aerosolized adenovirus-vectored vaccine as an alternative vaccine delivery method. Respir. Res..

[24] Buchbinder S.P., McElrath M.J., Dieffenbach C., Corey L. (2020). Use of adenovirus type-5 vectored vaccines: A cautionary tale. Lancet.

[25] Humphreys I.R., Sebastian S. (2018). Novel viral vectors in infectious diseases. Immunology.

[26] Vogel F.R., Sarver N. (1995). Nucleic acid vaccines. Clin. Microbiol. Rev..

[27] Park J.W., Lagniton P.N.P., Liu Y., Xu R.H. (2021). mRNA vaccines for COVID-19: What, why and how. Int. J. Biol. Sci..

[28] Smith T.R.F., Patel A., Ramos S., Elwood D., Zhu X., Yan J., Gary E.N., Walker S.N., Schultheis K., Purwar M. (2020). Immunogenicity of a DNA vaccine candidate for COVID-19. Nat. Commun..

[29] Knezevic I., Liu M.A., Peden K., Zhou T., Kang H.N. (2021). Development of mRNA Vaccines: Scientific and Regulatory Issues. Vaccines.

[30] Bettini E., Locci M. (2021). SARS-CoV-2 mRNA Vaccines: Immunological Mechanism and Beyond. Vaccines.

[31] Jackson N.A.C., Kester K.E., Casimiro D., Gurunathan S., DeRosa F. (2020). The promise of mRNA vaccines: A biotech and industrial perspective. NPJ Vaccines.

[32] Stone C.A., Rukasin C.R.F., Beachkofsky T.M., Phillips E.J. (2019). Immune-mediated adverse reactions to vaccines. Br. J. Clin. Pharmacol..

[33] Blumenthal K.G., Robinson L.B., Camargo C.A., Shenoy E.S., Banerji A., Landman A.B., Wickner P. (2021). Acute Allergic Reactions to mRNA COVID-19 Vaccines. JAMA.

[34] Tirado S.M., Yoon K.J. (2003). Antibody-dependent enhancement of virus infection and disease. Viral Immunol..

[35] Garber K. (2020). Coronavirus vaccine developers wary of errant antibodies. Nat. Biotechnol..

[36] Greinacher A., Thiele T., Warkentin T.E., Weisser K., Kyrle P.A., Eichinger S. (2021). Thrombotic Thrombocytopenia after ChAdOx1 nCov-19 Vaccination. N. Engl. J. Med..

[37] Sadoff J., Davis K., Douoguih M. (2021). Thrombotic Thrombocytopenia after Ad26.COV2.S Vaccination—Response from the Manufacturer. N. Engl. J. Med..

[38] Shimabukuro T., Nair N. (2021). Allergic Reactions Including Anaphylaxis After Receipt of the First Dose of Pfizer-BioNTech COVID-19 Vaccine. JAMA.

[39] Gao Q., Bao L., Mao H., Wang L., Xu K., Yang M., Li Y., Zhu L., Wang N., Lv Z. (2020). Development of an inactivated vaccine candidate for SARS-CoV-2. Science.

[40] Wu Z., Hu Y., Xu M., Chen Z., Yang W., Jiang Z., Li M., Jin H., Cui G., Chen P. (2021). Safety, tolerability, and immunogenicity of an inactivated SARS-CoV-2 vaccine (CoronaVac) in healthy adults aged 60 years and older: A randomised, double-blind, placebo-controlled, phase 1/2 clinical trial. Lancet Infect Dis..

[41] Zhang Y., Zeng G., Pan H., Li C., Hu Y., Chu K., Han W., Chen Z., Tang R., Yin W. (2021). Safety, tolerability, and immunogenicity of an inactivated SARS-CoV-2 vaccine in healthy adults aged 18–59 years: A randomised, double-blind, placebo-controlled, phase 1/2 clinical trial. Lancet Infect Dis..

[42] Han B., Song Y., Li C., Yang W., Ma Q., Jiang Z., Li M., Lian X., Jiao W., Wang L. (2021). Safety, tolerability, and immunogenicity of an inactivated SARS-CoV-2 vaccine (CoronaVac) in healthy children and adolescents: A double-blind, randomised, controlled, phase 1/2 clinical trial. Lancet Infect Dis..

[43] Tanriover M.D., Doğanay H.L., Akova M., Güner H.R., Azap A., Akhan S., Köse Ş., Erdinç F., Akalın E.H., Tabak Ö.F. (2021). Efficacy and safety of an inactivated whole-virion SARS-CoV-2 vaccine (CoronaVac): Interim results of a double-blind, randomised, placebo-controlled, phase 3 trial in Turkey. Lancet.

[44] Fadlyana E., Rusmil K., Tarigan R., Rahmadi A.R., Prodjosoewojo S., Sofiatin Y., Khrisna C.V., Sari R.M., Setyaningsih L., Surachman F. (2021). A phase III, observer-blind, randomized, placebo-controlled study of the efficacy, safety, and immunogenicity of SARS-CoV-2 inactivated vaccine in healthy adults aged 18–59 years: An interim analysis in Indonesia. Vaccine.

[45] Jara A., Undurraga E.A., González C., Paredes F., Fontecilla T., Jara G., Pizarro A., Acevedo J., Leo K., Leon F. (2021). Effectiveness of an Inactivated SARS-CoV-2 Vaccine in Chile. N. Engl. J. Med..

[46] Zhu F.C., Guan X.H., Li Y.H., Huang J.Y., Jiang T., Hou L.H., Li J.X., Yang B.F., Wang L., Wang W.J. (2020). Immunogenicity and safety of a recombinant adenovirus type-5-vectored COVID-19 vaccine in healthy adults aged 18 years or older: A randomised, double-blind, placebo-controlled, phase 2 trial. Lancet.

[47] Zhu F.C., Li Y.H., Guan X.H., Hou L.H., Wang W.J., Li J.X., Wu S.P., Wang B.S., Wang Z., Wang L. (2020). Safety, tolerability, and immunogenicity of a recombinant adenovirus type-5 vectored COVID-19 vaccine: A dose-escalation, open-label, non-randomised, first-in-human trial. Lancet.

[48] Zhu F., Jin P., Zhu T., Wang W., Ye H., Pan H., Hou L., Li J., Wang X., Wu S. (2021). Safety and immunogenicity of a recombinant adenovirus type-5-vectored COVID-19 vaccine with a homologous prime-boost regimen in healthy participants aged 6 years and above: A randomised, double-blind, placebo-controlled, phase 2b trial. Clin. Infect Dis..

[49] Halperin S.A., Ye L., MacKinnon-Cameron D., Smith B., Cahn P.E., Ruiz-Palacios G.M., Ikram A., Lanas F., Lourdes Guerrero M., Muñoz Navarro S.R. (2021). Final efficacy analysis, interim safety analysis, and immunogenicity of a single dose of recombinant novel coronavirus vaccine (adenovirus type 5 vector) in adults 18 years and older: An international, multicentre, randomised, double-blinded, placebo-controlled phase 3 trial. Lancet.

[50] Walsh E.E., Frenck R., Falsey A.R., Kitchin N., Absalon J., Gurtman A., Lockhart S., Neuzil K., Mulligan M.J., Bailey R. (2020). RNA-Based COVID-19 Vaccine BNT162b2 Selected for a Pivotal Efficacy Study. medRxiv.

[51] Polack F.P., Thomas S.J., Kitchin N., Absalon J., Gurtman A., Lockhart S., Perez J.L., Pérez Marc G., Moreira E.D., Zerbini C. (2020). Safety and Efficacy of the BNT162b2 mRNA COVID-19 Vaccine. N. Engl. J. Med..

[52] Kim H.W., Jenista E.R., Wendell D.C., Azevedo C.F., Campbell M.J., Darty S.N., Parker M.A., Kim R.J. (2021). Patients With Acute Myocarditis Following mRNA COVID-19 Vaccination. JAMA Cardiol..

[53] Mansour J., Short R.G., Bhalla S., Woodard P.K., Verma A., Robinson X., Raptis D.A. (2021). Acute myocarditis after a second dose of the mRNA COVID-19 vaccine: A report of two cases. Clin. Imaging.

[54] Fronza M., Thavendiranathan P., Chan V., Karur G.R., Udell J.A., Wald R.M., Hong R., Hanneman K. (2022). Myocardial Injury Pattern at MRI in COVID-19 Vaccine-associated Myocarditis. Radiology.

[55] Ghincea A., Ryu C., Herzog E.L. (2022). An Acute Exacerbation of Idiopathic Pulmonary Fibrosis After BNT162b2 mRNA COVID-19 Vaccination: A Case Report. Chest.

[56] Mumm T., Elbashir M. (2021). A Copd Exacerbation That Occurred after the Mrna COVID-19 Vaccine. Chest.

[57] Chen R.T., Hibbs B. (1998). Vaccine safety: Current and future challenges. Pediatr. Ann..

[58] Wu S., Huang J., Zhang Z., Wu J., Zhang J., Hu H., Zhu T., Zhang J., Luo L., Fan P. (2021). Safety, tolerability, and immunogenicity of an aerosolised adenovirus type-5 vector-based COVID-19 vaccine (Ad5-nCoV) in adults: Preliminary report of an open-label and randomised phase 1 clinical trial. Lancet Infect Dis..

[59] Zhou Z.H., Stone C.A., Jakubovic B., Phillips E.J., Sussman G., Park J., Hoang U., Kirshner S.L., Levin R., Kozlowski S. (2021). Anti-PEG IgE in anaphylaxis associated with polyethylene glycol. J. Allergy Clin. Immunol. Pract..

[60] Zhao J., Zhao J., Perlman S. (2010). T cell responses are required for protection from clinical disease and for virus clearance in severe acute respiratory syndrome coronavirus-infected mice. J. Virol..

[61] Channappanavar R., Fett C., Zhao J., Meyerholz D.K., Perlman S. (2014). Virus-specific memory CD8 T cells provide substantial protection from lethal severe acute respiratory syndrome coronavirus infection. J. Virol..

[62] Tay M.Z., Poh C.M., Rénia L., MacAry P.A., Ng L.F.P. (2020). The trinity of COVID-19: Immunity, inflammation and intervention. Nat. Rev. Immunol..

[63] Zhu F.C., Hou L.H., Li J.X., Wu S.P., Liu P., Zhang G.R., Hu Y.M., Meng F.Y., Xu J.J., Tang R. (2015). Safety and immunogenicity of a novel recombinant adenovirus type-5 vector-based Ebola vaccine in healthy adults in China: Preliminary report of a randomised, double-blind, placebo-controlled, phase 1 trial. Lancet.

[64] Buchbinder S.P., Mehrotra D.V., Duerr A., Fitzgerald D.W., Mogg R., Li D., Gilbert P.B., Lama J.R., Marmor M., Del Rio C. (2008). Efficacy assessment of a cell-mediated immunity HIV-1 vaccine (the Step Study): A double-blind, randomised, placebo-controlled, test-of-concept trial. Lancet.

[65] Haas E.J., Angulo F.J., McLaughlin J.M., Anis E., Singer S.R., Khan F., Brooks N., Smaja M., Mircus G., Pan K. (2021). Impact and effectiveness of mRNA BNT162b2 vaccine against SARS-CoV-2 infections and COVID-19 cases, hospitalisations, and deaths following a nationwide vaccination campaign in Israel: An observational study using national surveillance data. Lancet.

[66] Mittelman M., Magen O., Barda N., Dagan N., Oster H.S., Leader A., Balicer R. (2022). Effectiveness of the BNT162b2mRNA COVID-19 vaccine in patients with hematological neoplasms in a nationwide mass vaccination setting. Blood.

[67] Walsh E.E., Frenck R.W., Falsey A.R., Kitchin N., Absalon J., Gurtman A., Lockhart S., Neuzil K., Mulligan M.J., Bailey R. (2020). Safety and Immunogenicity of Two RNA-Based COVID-19 Vaccine Candidates. N. Engl. J. Med..

[68] Tauzin A., Nayrac M., Benlarbi M., Gong S.Y., Gasser R., Beaudoin-Bussières G., Brassard N., Laumaea A., Vézina D., Prévost J. (2021). A single dose of the SARS-CoV-2 vaccine BNT162b2 elicits Fc-mediated antibody effector functions and T cell responses. Cell Host Microbe..

[69] Collier A.Y., McMahan K., Yu J., Tostanoski L.H., Aguayo R., Ansel J., Chandrashekar A., Patel S., Apraku Bondzie E., Sellers D. (2021). Immunogenicity of COVID-19 mRNA Vaccines in Pregnant and Lactating Women. JAMA.

[70] World Health Organization Assessing the Programmatic Suitability of Vaccine Candidates for WHO Prequalification. https://apps.who.int/iris/bitstream/handle/10665/148168/WHO_IVB_14.10_eng.pdf;jsessionid=2B8144B8E176C5D64BCD778FA63C3110?sequence=1.

[71] Feikin D.R., Higdon M.M., Abu-Raddad L.J., Andrews N., Araos R., Goldberg Y., Groome M.J., Huppert A., O’Brien K.L., Smith P.G. (2022). Duration of effectiveness of vaccines against SARS-CoV-2 infection and COVID-19 disease: Results of a systematic review and meta-regression. Lancet.

[72] Nordström P., Ballin M., Nordström A. (2022). Risk of infection, hospitalisation, and death up to 9 months after a second dose of COVID-19 vaccine: A retrospective, total population cohort study in Sweden. Lancet.

[73] Andrews N., Stowe J., Kirsebom F., Toffa S., Rickeard T., Gallagher E., Gower C., Kall M., Groves N., O’Connell A.M. (2022). COVID-19 Vaccine Effectiveness against the Omicron (B.1.1.529) Variant. N. Engl. J. Med..

[74] Widge A.T., Rouphael N.G., Jackson L.A., Anderson E.J., Roberts P.C., Makhene M., Chappell J.D., Denison M.R., Stevens L.J., Pruijssers A.J. (2021). Durability of Responses after SARS-CoV-2 mRNA-1273 Vaccination. N. Engl. J. Med..

[75] Zhang J., He Q., An C., Mao Q., Gao F., Bian L., Wu X., Wang Q., Liu P., Song L. (2021). Boosting with heterologous vaccines effectively improves protective immune responses of the inactivated SARS-CoV-2 vaccine. Emerg. Microbes Infect..

